# Outcome of transanal advancement flap repair (TAFR) as salvage therapy after failed ligation of intersphincteric fistula tract (LIFT)—A proposed algorithm after failed LIFT

**DOI:** 10.1111/codi.70199

**Published:** 2025-08-07

**Authors:** E. Ergüder, C. Verkade, C. Ersak, J. Y. van Oostendorp, I. J. M. Han‐Geurts, D. K. Wasowicz, D. D. E. Zimmerman

**Affiliations:** ^1^ Department of Surgery Elisabeth‐TweeSteden Hospital Tilburg The Netherlands; ^2^ Department of Surgery Ankara Etlik City Hospital Ankara Turkey; ^3^ Department of Surgery Kızılcahamam State Hospital Ankara Turkey; ^4^ Department of Surgery Amsterdam UMC Amsterdam The Netherlands; ^5^ Department of Surgery Proctos Kliniek Bilthoven The Netherlands

**Keywords:** anal fistula, complex anal fistula, ligation of the intersphincteric fistula tract (LIFT), sphincter‐preserving procedure, Transanal advancement flap repair (TAFR)

## Abstract

**Aim:**

To assess the outcomes of transanal advancement flap repair (TAFR) following failed ligation of intersphincteric fistula tract (LIFT) treatment for cryptoglandular anal fistulas, with a focus on healing and functional results.

**Methods:**

This retrospective observational cohort study included patients who underwent TAFR after a failed LIFT procedure for cryptoglandular perianal fistulas at two Dutch referral centers between 2015 and 2023. Pre‐ and postoperative continence data were gathered prospectively, while long‐term outcomes, including success rates and faecal continence, were evaluated using the Faecal Incontinence Severity Index (FISI) through questionnaires distributed in 2023.

**Results:**

Of the 129 patients who underwent LIFT, 90 (70%) experienced failure of the procedure. A total of 24 patients, including those referred from other centres after failed LIFT, underwent TAFR, with a success rate of 71% (17 out of 24). No significant differences in faecal continence were observed between preoperative and postoperative FISI scores. Body mass index (BMI) was significantly associated with healing outcomes (*p* < 0.05), indicating that patients with a lower BMI had higher healing rates.

**Conclusion:**

TAFR following failed LIFT surgery shows healing results equivalent to those of initial fistula repairs, with obesity emerging as a potential predictor of healing success. These findings support current guideline recommendations favouring TAFR as the preferred salvage procedure after failed LIFT.

**What does this paper add to the literature?**

While multiple studies have investigated the success rates of surgical techniques for perianal fistulas, this study addresses the lack of data on TAFR as a salvage treatment after failed LIFT. It demonstrates a 71% success rate, providing new evidence to support clinical decision‐making strategies in managing recurrent perianal fistulas.

## INTRODUCTION

Treatment of transsphincteric cryptoglandular anal fistulas remains one of the most significant challenges in our field. According to the current guideline of the European Society of Coloproctology (ESCP) [1], both transanal advancement flap repair (TAFR) and ligation of the intersphincteric fistula tract (LIFT) are recommended as the preferred sphincter‐preserving techniques for the treatment of *primary* transsphincteric perianal fistulas that pass through the upper or middle portion of the external anal sphincter. Furthermore, the guideline explicitly states that when a fistula is amenable to LIFT, this procedure is the preferred choice. Despite the relatively favourable outcomes reported for LIFT, it is not always successful. In concordance with ESCP guidelines, in failed cases a salvage TAFR can be performed.

Recent ESCP guidelines recommend LIFT as the first‐choice treatment due to its low morbidity, short healing time, good functional outcomes in patients with fistulas that meet specific anatomical criteria [1]. A fistula should cross the intersphincteric space relatively directly without significant complexity (e.g. branching or horseshoeing), and the intersphincteric space must be sufficiently delineated, with an intact anal sphincter (IAS) and a small internal opening. Additionally, adequate space cephalad to the fistula is required to allow complete dissection without risking damage to the rectal wall or vagina, making some high tracts unsuitable for LIFT.

Despite its recommendation in clinical guidelines as the preferred salvage procedure following failed LIFT, the evidence base for TAFR in this specific context remains limited [1]. Robust data supporting its efficacy are scarce, with current guidelines relying primarily on clinical experience and expert consensus rather than extensively published research. This gap shows the need for further investigation into the success rates and outcomes of TAFR post‐LIFT failure to refine clinical decision‐making and improve patient care.

This study aimed to assess the healing and functional outcomes of TAFR performed as a salvage procedure after failed LIFT. Although no direct comparison was conducted with primary TAFR cohorts, our results are discussed in the context of previously reported outcomes for primary fistula repair, including both healing rates and functional outcomes such as continence, to explore whether salvage TAFR yields comparable effectiveness.

## METHODS

We conducted a retrospective observational cohort study using prospectively collected data from patients who underwent TAFR following failed LIFT for cryptoglandular perianal fistula between 2015 and 2023 in two Dutch referral centres: Elisabeth‐TweeSteden Hospital and Proctos Kliniek. Both institutions serve as tertiary and quaternary referral centres for complex perianal fistulas, with procedures performed by highly specialized surgeons. Only patients who underwent TAFR as salvage treatment after LIFT failure were included in the analysis.

As part of the standard treatment protocol, preoperative continence data were systematically collected prospectively for all patients. This data was extracted from electronic patient records and merged into a consolidated database. In May 2023, standardized questionnaires, including the Faecal Incontinence Severity Index (FISI), were distributed to all patients to collect long‐term data on recurrence rates, faecal continence and patient‐reported outcomes (The FISI quantifies the severity of faecal incontinence by scoring the frequency of incontinence to gas, mucus, liquid and solid stool, with higher scores indicating greater severity) [2]. The prospective database and the present study were approved by the Medical Ethical Committee (MEC no. 2022‐33).

Demographic characteristics (age and sex), body mass index (BMI), American Society of Anaesthesiologist (ASA) scores, smoking status, seton placement following the LIFT procedure, seton drainage duration, follow‐up duration and success rates were collected from the database.

### Inclusion criteria

All patients who underwent TAFR after failed LIFT for cryptoglandular fistulas between 2015 and 2023 were included. This included both patients treated at our own centre and those referred to us with MRI‐confirmed transsphincteric recurrences (referred to here as *full‐blown* recurrences, as opposed to more superficial recurrences limited to the intersphincteric incision site) following LIFT.

### Exclusion criteria

Patients were excluded if they had undergone initial surgical procedures other than LIFT, including drainage, fistulotomy, fistulectomy, laser therapy, Permacol paste application or primary TAFR. Additionally, patients with Crohn's disease or Hidradenitis Suppurativa were excluded from the study.

**FIGURE 1:**
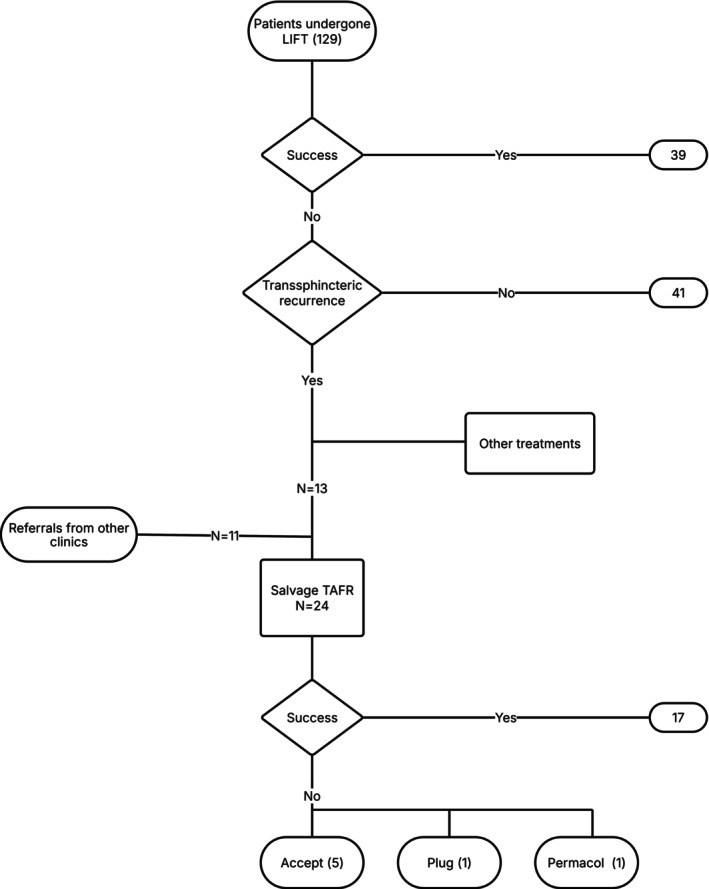
Flowchart illustrating the outcomes of patients who underwent LIFT.

The selection process is illustrated in Figure 1.

### Surgical procedure

**FIGURE 2:**
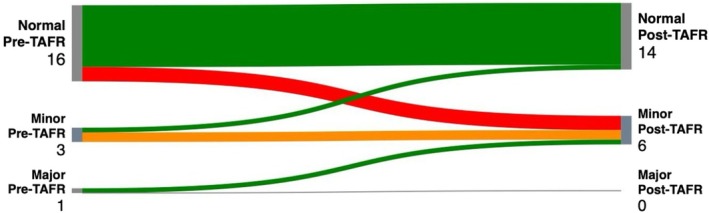
Sankey diagram of individual FISI scores preoperatively and postoperatively. FISI score; equal to zero is ‘Normal’, 1–30 is ‘Minor’, higher than 30 refers ‘Major’.

The TAFR procedure was performed according to established techniques, as previously described in the ESCP trainee video vignette: Transanal advancement flap repair; step‐by‐step guide for trainees [3]. After induction of general anaesthesia, intravenous prophylactic antibiotics were administered, consisting of metronidazole (500 mg) and cefuroxime (1500 mg). The external opening of the fistula was excised using microcautery (fistulectomy), and the fistulous tract was excised through this opening up to the border of the external anal sphincter. To expose the internal opening of the fistula, a Lone star retractor (Lone star Retractor System, Lone Star Medical Products®, Inc. Houston, TX, USA) was used. The crypt‐bearing tissue surrounding the internal opening, along with the overlying anoderm, was excised. The fistulous tract was then cored out of the sphincters, and the defect in the internal anal sphincter was closed with absorbable sutures. A flap consisting of mucosa, submucosa and a portion of the most superficial fibres of the internal anal sphincter was raised from the level of the dentate line and mobilized proximally over a distance of 4–6 cm. This flap was subsequently advanced and sutured to the neodentate line using absorbable sutures [4, 5]. To visually represent individual variations in continence status from preoperative assessment to follow‐up, Sankey diagrams were used (Figure 2). Complete clinical healing, meaning absence of discharge and external opening on examination, was confirmed during follow‐up visits. MRI was not routinely used to confirm healing post‐operatively.

### Statistical analysis

Statistical analyses were performed using IBM SPSS Statistics version 23.0 (IBM Corp, Armonk, NY, USA). Continuous variables were evaluated for normality using the Shapiro–Wilk test. Continuous variables including age, body mass index (BMI) and seton duration were presented as median and range (min‐max). Comparisons between healed and unhealed groups were conducted using the Mann–Whitney *U* test. Categorical variables such as smoking status and the presence of seton were reported as numbers and analysed using Fisher's exact test. ASA scores were compared using the chi‐square test. Pre‐ and post‐operative FISI scores were compared using the Wilcoxon signed‐rank test, and results were reported as median. *p*‐value less than 0.05 was considered statistically significant.

## RESULTS

A total of 129 patients underwent the LIFT procedure due to cryptoglandular fistula. The procedure was successful in 39 patients (30%), while the remaining 90 patients experienced persistent fistulas. These recurrences were classified as intersphincteric in 41 patients (32%) and transsphincteric in 49 patients (38%). Among patients with transsphincteric recurrence, various salvage treatments were applied following shared decision‐making between the surgical team and the patient, based on clinical characteristics, preferences and surgical feasibility. Thirteen of these patients underwent TAFR, while 28 patients received alternative treatments (e.g. laser ablation of the fistula tract (LAFT), redo‐ligation of the intersphincteric fistula tract (re‐LIFT), ligation of the intersphincteric fistula tract using a Permacol mesh (Bio‐LIFT), fistula plug, Permacol paste injection, fistulotomy or seton placement) and 8 patients were lost to follow‐up.

In addition, 11 patients with MRI‐confirmed full‐blown transsphincteric recurrences were referred from other centres for salvage TAFR. Together with the 13 patients treated at our own centre, a total of 24 patients underwent salvage TAFR and were included in this analysis. Seventeen of these 24 (71%) TAFR procedures were successful. Of the 7 patients with persistent fistula after TAFR, subsequent management included acceptance of the condition (*n* = 5), treatment with a fistula plug (*n* = 1) or Permacol injection (*n* = 1) (Figure 1).

A total of 24 patients (male 15 (63%), female 9 (37%), median age 45.8 years [range 23–81]) were included in the analysis of the present study. The median follow‐up period was 6 months (range 1–39), with a median BMI of 27.9 kg/m^2^ (range 20.6–40.7) (Table 1).

**TABLE 1:** Descriptive data of the patients with TAFR after LIFT (*n* = 24).

	Healed (*n* = 17)	Unhealed (*n* = 7)	*p* values
Age (years), median (range)	49.5 (31.8–81.1)	47 (41–62.7)	0.975
Gender			
Male	13	2	
Female	4	5	
BMI, median (range)	27.9 (22.2–31.2)	31.2 (24.1–31.4)	0.031*
ASA, *n*			0.205
1	9	2	
2	8	4	
3	‐	1	
Smoking, *n*			1
Yes	4	1	
No	13	6	
Seton present?, *n*			0.393
Yes	11	3	
No	6	4	
Seton duration (days), median (range)	127 (46–795)	182 (126–331)	0.586

* *p* < 0.05.

The continence status of patients with recurrent complex fistulas after failed LIFT surgery was evaluated using the FISI score. No significant difference was observed between medians of the preoperative and postoperative FISI scores (*p* = 0.183).

Due to the limited sample size, statistical power was insufficient to detect small differences in continence outcomes. To visualize individual changes in continence status, a Sankey diagram illustrates the continence outcomes before and after surgery (Figure 2).

Upon analysis, BMI was the only parameter significantly associated with fistula healing (*p* = 0.031). Specifically, patients with a lower BMI had a statistically significant higher likelihood of achieving healing compared to those with a higher BMI (Table 1).

However, no statistically significant associations were observed between sex, smoking status, ASA scores and healing outcomes (*p* > 0.05). Furthermore, when assessing the relationship between seton placement and healing, no statistically significant difference was found between patients who received a seton and those who did not (*p* > 0.05) (Table 1).

## DISCUSSION

In this study, we demonstrated that TAFR is a feasible and effective salvage option following LIFT failure. Following LIFT failure, TAFR is recommended as a salvage procedure, as endorsed by the ESCP guidelines [1]. However, intersphincteric recurrences, which may occur in up to 45.5% of cases, must first be ruled out. Although the numbers are limited, our study supports TAFR as a viable and safe salvage option, demonstrating satisfactory outcomes with healing rates and functional results comparable to those reported for primary TAFR procedures [6].

Notably, the healing rates after LIFT observed in our study are lower than those commonly reported in the literature. A recent meta‐analysis estimated an average healing rate of 69.1% after LIFT [6]; though some series have reported lower healing rates [7, 8]. Several factors may contribute to this discrepancy. While unlikely, surgical quality across centres could be a factor, as both centres openly reported all cases, including early‐stage procedures, potentially reflecting a learning curve effect. Additionally, patient selection bias may play a role, as both centres serve as tertiary and quaternary referral centres in the Netherlands and thus receive complex and recurrent cases. Given that operative techniques were aligned with international standards and referenced against animal models, it is unlikely that technical deficiencies explain the lower healing rates. Further investigation into whether outcomes improved over time with continued procedural experience is warranted.

Another critical factor to consider is the rigorous follow‐up methodology employed in our study. The meticulous post‐operative monitoring ensured accurate identification of outcomes, which may explain the discrepancy between our results and those reported in studies with shorter follow‐up durations. It is essential to take this into account when interpreting long‐term data, as variations in follow‐up length can significantly influence reported success rates. Additionally, functional outcomes require further investigation, and we encourage other researchers to publish long‐term results using objective functional outcome measures. Although our follow‐up duration is limited, this reflects the constraints of the retrospective dataset and cannot be extended. Nevertheless, previous studies have shown that most failures after transanal advancement flap repair occur within the early postoperative period and that healing rates tend to stabilize thereafter [9]. We therefore believe that our follow‐up period provides a reliable estimate of healing outcomes. However, this does not affect the primary focus of the present study, which concerns the outcomes of salvage therapy after LIFT failure, rather than the effectiveness of LIFT as a primary procedure.

When LIFT fails, the success rate of the subsequent TAFR procedure may be influenced by various factors. Interestingly, in previous research evaluating predictive factors for successful LIFT procedures in transsphincteric fistulas, the presence of a drainage seton placed before LIFT did not affect the outcomes [10]. However, after an initial TAFR fails, a repeat TAFR can achieve a success rate of 69%, leading to an overall cumulative success rate of approximately 90% [11]. Although some authors have proposed that preoperative seton drainage might improve TAFR outcomes, this finding has not been consistently confirmed in the literature. Furthermore, existing studies have not clearly established whether the potential benefit comes specifically from seton placement itself or simply from the extended time interval between procedures [4, 12]. Based on our limited data, we tentatively conclude that neither the insertion of a seton nor the duration for which the seton remained in place after a failed LIFT significantly affected the success rate of subsequent TAFR procedures (78%–67%) A recent long‐term follow‐up study conducted in two reference centres in the Netherlands showed that seton application increased the success rate of primary LIFT surgery [5]. On the other hand, similar to our findings, Mitalas et al. and Van Koperen et al. found that seton applications had no effect on the success rate of primary TAFR procedures (63%–67% *p* = 0.40; 76%–82% *p* = 0.53) [6, 12]. Of course, we have to consider the ‘seton paradox’ [13], in which fistulas with a lot of inflammation and/or granulation tissue (i.e. more difficult to treat) are more likely to undergo seton drainage, yet yield similar results to fistulas that do not display this.

Additionally, long‐term seton placement can provide a conservative treatment option for patients preferring to delay definitive surgery, ensuring adequate drainage and infection control while maintaining individual treatment flexibility. Despite conflicting evidence, we concur with the ESCP guidelines that a preparatory seton is not mandatory and should be considered based on patient comfort, excessive inflammation or suppuration.

Our findings suggest that a higher BMI may be associated with lower healing rates after salvage TAFR. In our cohort, patients with unsuccessful healing had a median BMI of 31.2 kg/m^2^, which is higher than other studies that evaluated the success rate of LIFT or TAFR (Arajuo et al. 26.4 kg/m^2^) [14]. However, when the cohort was stratified by a BMI cutoff of 30 kg/m^2^, the difference between groups did not reach statistical significance (Fisher's exact test, *p* = 0.167), despite a clear numerical disparity in healing rates (81.3% in BMI <30 vs. 50% in BMI ≥30). This discrepancy may be attributed to the limited sample size, which could have reduced the power of the categorical analysis to detect significance. Nevertheless, the observed trend aligns with the continuous analysis and suggests a potential adverse effect of higher BMI on healing, warranting further investigation in larger, adequately powered studies.

While a definitive link between high BMI and LIFT failure has not been established, our data support the hypothesis that elevated BMI might be associated with decreased success rates of TAFR following LIFT failure [10]. Interestingly, Yang et al. reported that high BMI is a risk factor for the development of anal fistulas [15]. The exact mechanism remains unclear, but obesity‐associated comorbidities, such as impaired wound healing and chronic inflammation, may contribute. However, ASA scores, which typically reflect comorbidity burden, did not show a significant difference between healed and non‐healed groups, nor did smoking status, despite previous reports suggesting an association [16, 17].

The Sankey diagram (Figure 2) illustrates continence variations before and after TAFR. Patients were categorized as having ‘normal’, ‘minor’ or ‘major’ incontinence and the visual flow demonstrates that TAFR did not result in substantial continence deterioration. Most patients who had normal continence preoperatively remained continent, while those with minor or major incontinence showed either stability or improvement postoperatively. Unlike traditional analyses relying on median or mean continence scores, this approach provides a more granular view of individual patient trajectories. Thus, our data indicate that TAFR, when conducted after LIFT, maintains continence and does not exacerbate pre‐existing problems for most patients.

This study is limited by its retrospective design and relatively small sample size, particularly within specific subgroups such as transsphincteric recurrences. The modest number of cases collected over a prolonged period may increase susceptibility to random variation and selection bias and may limit generalizability. In addition, changes in clinical practice over time could have influenced diagnostic and therapeutic strategies. Although efforts were made to mitigate confounding through careful case selection and standardized data extraction, the retrospective design limits our ability to fully account for factors such as disease severity, prior interventions and perioperative decision‐making. The absence of a control group and the limited follow‐up period further constrain the interpretation of long‐term outcomes. Nonetheless, given the rarity and complexity of this patient population, our findings offer valuable insights and provide a foundation for future prospective research with larger cohorts and longer follow‐up durations to validate these results and refine treatment strategies.

**FIGURE 3:**
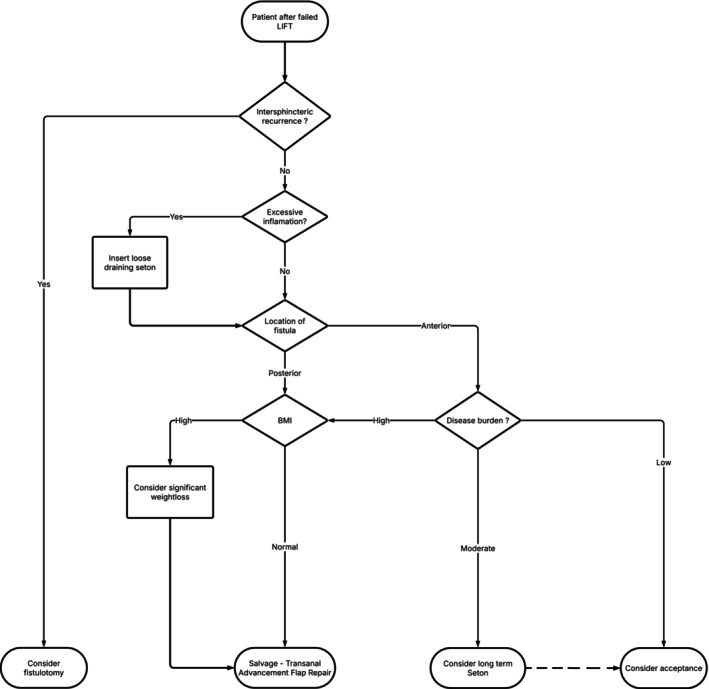
Proposed stepwise treatment algorithm based on expert opinion and clinical experience.

Based on our findings, we will continue to apply this stepwise treatment algorithm in clinical practice for managing persistent disease after failed LIFT, as our results do not indicate a need for modification at this stage (Figure 3). First, a thorough evaluation of the fistula tract and local inflammation is essential. In most cases, imaging will not be necessary for this. If an intersphincteric recurrence is identified, fistulotomy may be an option for patients with intact continence. If a high transsphincteric fistula tract persists with inflammation, a seton should be placed to ensure adequate drainage before definitive surgery. Once inflammation subsides, fistula management should be individualized based on fistula location (posterior vs. anterior), disease burden and patient factors (e.g. BMI and continence status). For posterior fistulas, salvage TAFR is recommended as the first‐line option. In cases of high BMI, weight loss should be considered to improve surgical outcomes before definitive salvage TAFR. For anterior fistulas, management is more challenging. Formal advancement flap repair is often discouraged, particularly in female patients [18]. This presents a major challenge in determining the optimal approach for female patients with anterior full‐blown recurrences. Alternative strategies such as LAFT may be considered, although with limited success rates. Therefore, for anterior fistulas, management varies depending on disease severity. For low‐burden cases, long‐term seton placement or acceptance of the condition may be viable options. In cases of high disease burden, intervention is warranted. This structured approach ensures individualized treatment strategies, optimizing outcomes after a failed LIFT procedure.

## CONCLUSION

TAFR is an effective and safe salvage therapy for complex cryptoglandular fistulas after failed LIFT.

## AUTHOR CONTRIBUTIONS

**E. Ergüder:** Writing – original draft; data curation; formal analysis; investigation; validation; visualization; methodology; project administration. **C. Verkade:** Investigation; resources. **C. Ersak:** Formal analysis; visualization; writing – original draft; methodology; validation. **J. Y. van Oostendorp:** Writing – review and editing; resources; investigation; methodology; validation. **I. J. M. Han‐Geurts:** Resources. **D. K. Wasowicz:** Data curation; writing – review and editing; investigation; resources. **D. D. E. Zimmerman:** Writing – review and editing; writing – original draft; resources; conceptualization; investigation; visualization; supervision.

## FUNDING INFORMATION

The authors have no funding to declare.

## CONFLICT OF INTEREST STATEMENT

The authors declare no conflict of interest.

## ETHICS STATEMENT

The study was approved by the Medical Ethical Committee Brabant (MEC nr 2022‐33).

## INFORMED CONSENT

Informed consent was obtained from all individuals.

## Data Availability

The data that support the findings of this study are available from the corresponding author upon reasonable request.
